# 25(OH)D Status of Elite Athletes with Spinal Cord Injury Relative to Lifestyle Factors

**DOI:** 10.3390/nu8060374

**Published:** 2016-06-17

**Authors:** Kelly Pritchett, Robert Pritchett, Dana Ogan, Phil Bishop, Elizabeth Broad, Melissa LaCroix

**Affiliations:** 1 Department of Nutrition, Exercise, and Health Sciences, Central Washington University, 400 E. University Way, Ellensburg, WA 98926, USA; pritchettr@cwu.edu (R.P.); ogand@cwu.edu (D.O.); 2 Department of Kinesiology, the University of Alabama, P.O. Box 870312, Tuscaloosa, AL 35487, USA; pbishop@bamaed.ua.edu; 3 US Olympic Committee, 2800 Olympic Parkway, Chula Vista, CA 91915, USA; elizabeth.broad@usoc.org; 4 Canadian Sport Institute Pacific, 6111 River Rd, Richmond, BC V7C 0A2, Canada; mlacroix@csipacific.ca

**Keywords:** 25(OH)D, sun exposure, spinal cord injuries, athletes

## Abstract

**Background:** Due to the potential negative impact of low Vitamin D status on performance-related factors and the higher risk of low Vitamin D status in Spinal Cord Injury (SCI) population, research is warranted to determine whether elite athletes with SCI have sufficient 25(OH)D levels. The purposes of this study were to examine: (1) the seasonal proportion of vitamin D insufficiency among elite athletes with SCI; and (2) to determine whether lifestyle factors, SCI lesion level, and muscle performance/function are related to vitamin D status in athletes with SCI. **Methods:** Thirty-nine members of the Canadian Wheelchair Sports Association, and the US Olympic Committee Paralympic program from outdoor and indoor sports were recruited for this study. Dietary and lifestyle factors, and serum 25(OH)D concentrations were assessed during the autumn (October) and winter (February/March). An independent t-test was used to assess differences in 25(OH)D status among seasons, and indoor and outdoor sports in the autumn and winter, respectively. **Results:** Mean ± SD serum 25(OH)D concentration was 69.6 ± 19.7 nmol/L (range from 30 to 107.3 nmol/L) and 67.4 ± 25.5 nmol/L (range from 20 to 117.3 nmol/L)in the autumn and winter, respectively. In the autumn, 15.4% of participants were considered vitamin D deficient (25(OH)D < 50 nmol/L) whereas 51.3% had 25(OH)D concentrations that would be considered insufficient (<80 nmol/L). In the winter, 15.4% were deficient while 41% of all participants were considered vitamin D insufficient. **Conclusion:** A substantial proportion of elite athletes with SCI have insufficient (41%–51%) and deficient (15.4%) 25(OH)D status in the autumn and winter. Furthermore, a seasonal decline in vitamin D status was not observed in the current study.

## 1. Introduction

The Third National Health and Nutrition Examination Survey (NHANES III) determined that over 77% of Americans are considered vitamin D insufficient [1]. Vitamin D is well known for its role in bone health [2], but recent research has linked vitamin D to other important processes in the body including: hormone synthesis, signaling gene response, immunity, protein synthesis, and cell turnover [3,4,5,6,7]. These rates of insufficiency (25(OH)D < 80 nmol/L), together with the essential metabolic properties of vitamin D, have led researchers to examine the influence of vitamin D not only on bone health, but also on physical performance and injury in athletes.

Vitamin D receptors have recently been identified in skeletal muscle, which has led to further examination of the influence of vitamin D on athletic performance [7]. Therefore, the risk of vitamin D insufficiency among athletes has received growing interest. In the last decade, researchers have examined 25(OH)D (criterion measure for vitamin D concentration levels) among various groups of athletes including: gymnasts [8], runners [4,9,10], rugby players [10], and jockeys [11] and have suggested that athletes are at a high risk for vitamin D insufficiencies [7]. Blood 25(OH)D concentrations are largely dependent on geographical location (latitude) and type of sport (indoor *vs.* outdoor) [3,7].

Individuals with spinal cord injury (SCI) may be at increased risk for vitamin D insufficiency due to inadequate diet [12], anticonvulsant medications, and reduced sunlight exposure [13]. The ability of individuals with spinal cord injury to go outdoors and synthesize vitamin D from sunlight may be limited due to decreased functional mobility, impaired thermoregulation, amount of skin exposed, season of the year, and latitude. Oleson *et al.* (2006) found that 65% of patients with acute SCI, and 81% of patients with chronic SCI had insufficient 25(OH)D levels in the summer, despite living in Birmingham, AL (33°N) [13]. These rates increased to 84% and 96% in the winter months [13]. Hummel *et al.* (2012) found that 39% of individuals (*n* = 62) with chronic SCI had insufficient 25(OH)D regardless of supplementation use [14]. Furthermore, Hummel *et al.* (2012) suggested that optimal serum 25(OH)D levels for SCI may be higher than in an able bodied population due to an increased 25(OH)D needed to suppress parathyroid hormone (PTH) release and absorb calcium [14]. Considering individuals with SCI are already at high risk for osteoporosis [13,15,16], a low serum 25(OH)D puts them at an even greater risk for bone injuries and fractures given that the onset of bone loss occurs quickly post injury [13,14,17]. Although performance trials are limited in an athletic population, a number of studies support vitamin D’s indirect role in enhancing exercise performance [18,19,20,21,22], however recent studies do not support the benefits of vitamin D supplementation [23,24]. A recent meta-analysis suggested that vitamin D supplementation has a small, but positive, impact on muscle strength in healthy individuals [22]. Favorable 25(OH)D levels may reduce the risk of debilitating stress fracture among athletes, indirectly influencing performance through prevention of injury [25,26]. Finally, because vitamin D is used in numerous metabolic pathways, it has been suggested that the athlete may need a higher vitamin D status to ensure acceptable energy availability and storage [27].

Knowledge regarding the vitamin D status of athletes with SCI is lacking. Due to the potential negative impact of low vitamin D status on performance-related factors and the potentially higher risk of vitamin D insufficiency in SCI population, research is warranted to determine whether elite athletes with SCI have sufficient 25(OH)D levels. The purposes of this study were to examine (1) the seasonal proportion of vitamin D insufficiency among elite athletes with SCI; and (2) to determine whether lifestyle factors, SCI lesion level, and muscle performance/function are related to vitamin D status in athletes with SCI. Based on previous studies conducted in athletes [9,10], we hypothesized that a seasonal decline in 25(OH)D status will be observed from autumn to winter. In addition, rates of vitamin D insufficiency will be higher among elite athletes with SCI participating in indoor sports compared to those participating in an outdoor sport, which may be related to increased time spent indoors [13], decreased mobility, and vitamin/mineral deficiencies that are commonly a problem in individuals with SCI [12].

## 2. Methods

### 2.1. Subjects

Male and female athletes, ≥18 years old, were recruited via the sport medicine staff from The Canadian Wheelchair Sports Association, and the US Olympic Committee Paralympic programs. Athletes were screened for eligibility and then recruited via members of the sports medicine team. Only athletes eligible for participation were approached. Participating athletes with SCI were required to have an impairment of their spinal cord (e.g., spinal cord injury, spina bifida) and were selected from tennis, athletics, basketball and rugby. Exclusion criteria included athletes with a diagnosis of fat malabsorption, thyroid, kidney, or bone disease. Based on data from previous studies [13] using a mean of 37.4 ± 24.9 nmol/L, a power of 0.8 and alpha = 0.05, an a priori power analysis (G Power version 3.1) indicated the need for 40 participants. This study was approved by the Central Washington University (CWU) Human Subjects Review Committee (HSRC) (Project # H14114). Subjects provided written consent prior to participating in the study.

### 2.2. Study Design

A longitudinal, observational trial examined seasonal changes (summer, winter) in 25(OH)D status. Participants reported to the testing facility on two separate data collection sessions (involving 2 days), initially at the beginning of autumn (October 2014, for maximal cumulative sun exposure) and for a follow up in the winter (February/March 2015, for minimal cumulative sun exposure).

Descriptive characteristics including height, weight, injury level, and history of injury were recorded. Prior to testing, participants were asked to sustain normal hydration levels, and consume their usual breakfast for both testing sessions (autumn, winter), which was recorded and analyzed for macronutrient (carbohydrate, protein, and fat) content. In addition, participants were asked to complete a Diet and Lifestyle Questionnaire. Participants’ supplement routine was recorded for each trial to account for any changes.

The following measurements were collected:

### 2.3. 25(OH)D Assay

The 25(OH)D levels were assessed during each data collection session. Blood samples consisted of 5–10 drops of blood obtained from the fingertips using a lancet and sterile procedures. Drops of whole blood were pipetted onto blood spot cards. Blood spots were air dried for at least 30 min and sent in batches to a certified laboratory (ZRT Laboratory, Beaverton, OR, USA) for 25(OH)D assay. Blood spot assay has shown excellent correlation (*r* = 0.97; with a lower limit of detection of 1.9 ng/mL) with liquid chromatography/tandem mass spectrometry assay and has been suggested to provide a convenient alternative [28]. Results were compared to reference values (Table 1), and various other published 25(OH)D concentrations from studies conducted in healthy adults [9,29,30,31].

### 2.4. Diet and Lifestyle Questionnaire

Participants completed a self-administered questionnaire at each data collection session that focused on factors that could potentially influence vitamin D status including dietary intake, supplement use (vitamin D, multivitamin, and calcium), UVB exposure, sunscreen use, injuries and illness. Instructions were provided prior to administration of the questionnaire. The questionnaire was replicated, with permission, from Halliday *et al.* (2010) [9]. This frequency questionnaire addressed dietary intake, supplement use, UVB exposure, sunscreen use, injuries and illness. Participants rated how often they consumed vitamin D-containing foods and supplements (never or *<*1 per month, 1–3 per month, 1 per week, 2–4 per week, 5–6 per week, 1 per day, 2–3 per day, 4–5 per day, or 6 or more per day). In addition, the frequency of time spent outdoors (never or <10 min per week, 1–3 h per month, 1 h per week, 2–4 h per week, 5–6 h per week, 0.5–1 h per day or >2 h per day), time of day when sun exposure occurred (6–10 a.m., 10 a.m. to 2 p.m., 2–6 p.m.), tanning bed use (never or <10 min per week, 10–20 min per week, 20–30 min per week, 30–40 min per week, 40–50 min per week, 50–60 min per week or >60 min per week), sunscreen use (on 100 mm scale with left anchor of NEVER, and right anchor of ALWAYS), Sun protection factor (SPF) of sunscreen used, type clothing worn, and frequency of illness was self-reported [9]. Participants reported their responses based on their location during the previous three months.

The average vitamin D content of food listed in the questionnaire was calculated from the United States Department of Agriculture (USDA) national nutrient database for standard reference [32], and food labels as derived from Halliday *et al.* (2010). Daily average intakes, expressed in international units (IU), were calculated by “multiplying the frequency midpoint by the average” vitamin D content in each food or supplement [9].

### 2.5. Performance Tests

*The 20 m SPRINT* A self-regulated, warm up was matched with autumn, and winter trials. 20 m sprint tests were completed using 4 sets of timing lights (Brower Timing Systems, Draper, UT, USA) to measure 5 m, 10 m, and 20 m to splits. Where timing gates were not accessible, a hand held stopwatch was used to record time for the 20 m sprint. A 20 M sprint test is commonly used to assess anaerobic performance in athletes with SCI.

*Handgrip strength* was assessed using a handgrip dynamometer (model 68812 County Technology INC, Gays Mills, WI, USA) using the dominant hand first. The grip width was adjusted until the second joint of the participant’s forefinger was bent at 90 degrees during the grasp. The indicator was set to zero before each trial. In a seated position and relaxed, with their elbows at 90 degrees participants gripped the dynamometer with their dominant hand to exert full force without letting their arms touch their body or rest on any part of their wheelchair. These procedures were repeated twice on both the left and right hands, and the highest score was recorded for a sum of the two hands.

### 2.6. Data Analysis

Data were analyzed using IBM SPSS for Windows version 18.0 software (SPSS Inc, Chicago, IL, USA). Basic descriptive statistics (mean ± SD) were computed to describe the sample population, and to quantify 25(OH)D status in this population. Data were examined for normalcy using the Komogorov-Smirnov test for skewness and kurtosis. The results indicated a normal distribution, therefore means ± SD, and parametric tests were used to describe and examine the sample. Given there were no differences in 25(OH)D status and geographical location, USA and CAN athletes were analyzed as one group. Pearson *r* correlations were used to examine the relationship between 25(OH)D levels and vitamin D intake. Spearman rank correlations were used to assess the relationship between serum 25(OH)D concentrations and non-continuous variables, including frequency of intake of vitamin D–containing foods and supplements, leisure time spent outdoors, tanning bed use, and frequency of illness. A one-way repeated measures analysis of variance (ANOVA) was used to examine differences in 25(OH)D status based on level of lesion. A Tukey *post hoc* test was applied in the case of significant (*p* ≤ 0.05). A paired *t*-test was used to assess difference in 25(OH)D status among seasons, and indoor and outdoor sports in the autumn, and winter. A Pearson correlation was used to analyze associations between 25(OH)D and muscle function measures. Cohen’s d effect size was calculated for mean difference between indoor and outdoor sports in the autumn and winter, respectively. The alpha level was set at 0.05.

## 3. Results

Volunteers (height: 131.5 + 13.6 cm; weight: 59.5 + 13.5 kg; age: 27.7 + 6.5 years) (*n* = 39: 19 male, and 20 female; 1 African American, 1 White Hispanic, 3 Asian, and 30 Caucasian) from outdoor: tennis (*n* = 1) and athletics (track and field) (*n* = 14), and indoor sports: rugby (*n* = 12), and basketball (*n* = 12). All athletes with SCI were analysed in a single group, due to the small sample size which is typical for research examining athletes with SCI. Seven participants did not complete the winter testing session due to not being selected for the next team camp or travel. Therefore, only 32 athletes completed the final testing session in the winter. Baseline characteristics were not significantly different between dropouts and those who finished the study. Descriptive characteristics and seasonal 25(OH)D status organized by sport are displayed in Table 2.

### 3.1. The 25(OH)D Status

In the autumn, mean 25(OH)D concentrations averaged 69.6 ± 19.7 nmol/L (range 30 to 107.3 nmol/L). 15.4% *(n =* 6) of participants were considered vitamin D deficient (25(OH)D < 50 nmol/L) whereas 51.3% (*n* = 20) had 25(OH)D concentrations that would be considered insufficient (50–79.9 nmol/L). 6% of outdoor athletes were classified as deficient and 60% as insufficient for 25(OH)D status compared to 21% of indoor athletes classified as deficient and 46% insufficient. 33% of participants in both groups were classified as sufficient for 25(OH)D status. There was no significant difference (*p* = 0.19) in 25(OH)D among indoor and outdoor sports, respectively (indoor 66.3 ± 21.5 nmol/L; outdoor 74.8 ± 16.2 nmol/L; ES = 0.45).

In the winter, mean 25(OH)D concentrations averaged 67.4 ± 25.5 nmol/L (ranging from 20 to 117.3 nmol/L). There was no significant difference (*p* = 0.68) in Vitamin D status between autumn and winter (autumn: 69.6 ± 19.7 nmol/L, winter 67.4 ± 25.5) for the total group. 41% of all participants were considered vitamin D insufficient, while 15.4% were deficient. Furthermore, 6% of outdoor athletes were classified as deficient and 56% as insufficient for 25(OH)D status compared to 21% of indoor athletes classified as deficient and 30% insufficient. 23% of all participants in the winter were classified as sufficient. In addition, there was no significant difference (*p* = 0.75) in 25(OH)D among indoor and outdoor sports, respectively (indoor 66.1 ± 28.5 nmol/L; outdoor 69.8 ± 21.5 nmol/L; ES = 0.16).

For all participants, 25(OH)D concentration in the autumn was correlated with 25(OH)D concentrations in the winter (*r* = 0.59, *n* = 32, *p* < 0.001). Plasma 25(OH)D was not significantly different between sports team during autumn or winter (Table 2) or between gender in the autumn (*p* = 0.29) (females 73.1 ± 18.5; males 66.1 ± 21.2) or winter (*p* = 0.59) (females 65.2 ± 23.2; males 70.1 ± 28). Furthermore, the mean differences observed between 25(OH)D concentrations and level of lesion were not statistically different in the autumn (*p* = 0.15) and winter (*p* = 0.59), respectively (Table 3).

### 3.2. Dietary Intake of Vitamin D and Vitamin D Status

Frequency of consumption of vitamin D containing foods is displayed in Table 4. The average dietary intake of vitamin D from food sources was 121.1 ± 9.8 IU/day in the autumn, and 115 ± 12.25 IU/day in the winter. Milk consumption in the autumn and winter averaged 2 to 4 servings per week. In the autumn and winter, the estimated average frequency for multivitamin (MVI) intake was “never”. Vitamin D status was correlated with milk consumption in the autumn (*r* = 0.27, *p ≤* 0.05), and in the winter (*r* = 0.58, *p ≤* 0.05). However, no other correlations were found between 25(OH)D status and supplements, or vitamin D containing foods. In the winter, vitamin D status was correlated with calcium supplementation (*r* = 0.33, *p* = 0.04).

### 3.3. UVB Exposure and Vitamin D Status

Reported leisure time spent outdoors was significantly different across seasons (*p* ≤ 0.001) and averaged 5.5 ± 1.6 h/week^−1^ in the autumn, and 2 ± 1.5 h/week^−1^ winter, respectively. Leisure time spent outdoors was significantly (*r* = 0.41, *p* ≤ 0.05) correlated with vitamin D status in the autumn, but not in the winter (*r* = −0.02, *p* = 0.46). However, the time of day spent outdoors (daylight hours independent of hours spent outdoors), geographical location, sunscreen use, or SPF (sun protection factor) was not correlated with 25(OH)D status for either season.

### 3.4. Muscle Function and Vitamin D Status

Pearson correlations for 20 M sprint time and 25(OH)D were not significant during the autumn (*r* = −0.16, *p* = 0.17) or winter (*r* = −0.03, *p* = 0.45), or for handgrip strength and 25(OH)D during the autumn (*r* = 0.2, *p* = 0.14) or winter (*r* = 0.08, *p* = 0.35), respectively.

## 4. Discussion

Our findings suggest that a substantial proportion (41%–51%) of elite athletes with SCI have insufficient vitamin D status in the winter, and autumn. Although a seasonal decline in vitamin D status was not observed in the current study, a higher proportion of athletes were considered sufficient in the autumn compared to the winter (33% *vs.* 23%, respectively). Furthermore, there was no significant difference in 25(OH)D among indoor and outdoor sports. The proportion of vitamin D insufficiency observed in these athletes with SCI was lower than vitamin D insufficiency rates reported in a group of sedentary individuals with SCI (81%), and the general U.S. population (77%) from 2001 to 2004 [1,13]. These findings support other literature suggesting that high-risk athletes, such as indoor athletes and those who avoid peak daylight hours, should have 25(OH)D levels assessed annually [7,9]. Because vitamin D status may play a role in the development of osteoporosis [13,14,15], injury risk [13,14,17] and exercise performance [18,19,20,21,22,23,24], further research is warranted to appropriately identify serum 25(OH)D goal levels in athletes with SCI and to determine the magnitude of effect from vitamin D status on muscle strength and performance.

Similar to studies conducted in able-bodied athletes [9,10], 15% of our participants were considered vitamin D deficient while 51% had 25(OH)D concentrations that would be considered insufficient in the autumn. Furthermore, 6% of outdoor athletes with SCI were deficient compared to 21% of indoor athletes with SCI in the autumn. In contrast, a study conducted in Laramie, WY (41.3°N), found lower vitamin D insufficiency rates (12%) athletes (*n* = 41) after the summer months compared to the autumn [4]. Another study suggested that 73% of indoor athletes (gymnasts and dancers) were vitamin D insufficient despite living at favorable latitude for UVB exposure (Israel 31.8°N) [33].

Recent findings by Flueck *et al.* (2016) suggested that 73.2% of Paralympic athletes have insufficient/deficient 25(OH)D status [34]. Furthermore, 25(OH)D levels were significantly lower during the winter months [31], however it should be noted that the study was cross sectional in nature and did not examine the within subject change from autumn to winter. The authors concluded that athletes need to be tested for 25(OH)D status in the autumn, and again in the winter/early spring months as performed in the current study. Contrary to findings in able-bodied athletes [9,10], and in a group of sedentary individuals with SCI [13], a seasonal decline in vitamin D status was not observed in the current study. In the winter, comparable rates of vitamin D insufficiency (41%) and deficiency (15%) were observed in athletes with SCI.

In the current study, sunlight was the primary source of vitamin D during the summer months leading into autumn. Sun exposure significantly (*p* ≤ 0.001) decreased from autumn to winter (5.5 ± 1.6 h/week^−1^ in autumn, and 2 ± 1.5 h/week^−1^ in winter). Furthermore, time spent outdoors was associated with vitamin D status in the autumn, but not in the winter. Oleson *et al.* (2006) found that patients with chronic SCI had sub therapeutic 25(OH)D levels in the summer despite living in Birmingham, AL (33°N) and these rates increased to 84% and 96% in the winter months [13]. It should be noted that heat and humidity may create a barrier for individuals with SCI to go outdoors during the summer months due to difficulties with thermoregulation. Similar to previous research, geographical location (latitude) and gender did not appear to be the major risk factors for vitamin D insufficiency in athletes with SCI [9,10]. As seen in able-bodied indoor athletes, lack of sun exposure may increase the risk for vitamin D insufficiency [7].

The average vitamin D intake observed in the current study did not meet the minimum Dietary Recommended Intake (DRI; 600 IU/day for adults 18–70 years of age) to prevent a clinical vitamin D deficiency [21]. According to National Health and Nutrition Examination Survey (NHANES) data, only 9.4% of individuals with a disability (*n* = 11,811) meet the recommendations for vitamin D intake from food alone [35]. Krempien *et al.* (2011) suggested that vitamin D intake from food alone was inadequate in a group of elite Canadian athletes with SCI (Males 87 ± 66 IU/day, Females 166 ± 130 IU/day) [12]. In addition, the estimated prevalence of inadequate intake was the highest for vitamin D when compared to other vitamins or minerals [12]. However, it should be noted that vitamin D intake from foods was the major source of vitamin D in the winter months.

Finally, it has been suggested that vitamin D supplementation may be necessary to maintain adequate levels during the winter even in athletes who spend ample time outdoors [7,36], however this has yet to be examined in athletes with SCI. Furthermore, Storlie *et al.* (2011) suggested that supplementation with 1000 IU/day of vitamin D was not enough to prevent seasonal decline of vitamin D status in male athletes [10]. Hummel *et al.* (2012) found that 39% of individuals (*n* = 62) with chronic SCI had suboptimal 25(OH)D levels, although a large majority of subjects were taking vitamin D supplements [14]. Therefore, vitamin D supplementation protocols for athletes with SCI needs to be established.

Athletes with SCI exemplify a fascinating group to examine due to the diversity of physical impairment resulting in a variety of physiological abilities [37]. In the current study, measures of muscle function/performance (handgrip strength and sprint time) were not associated with 25(OH)D status during the autumn or winter. Muscle wasting below the level of the lesion (which reflects both level and completeness of injury) may hinder the demonstration of any possible relationship between 25(OH)D levels and muscle strength tests in individuals with SCI. We can only speculate that the ability to detect a change in performance measures may be limited given that the subjects in the current study are highly trained, elite athletes with SCI. In a study by Barbonetti *et al.* (2016), lower 25(OH)D levels showed a significant independent association with poorer physical function outcomes, after adjusting for several confounders in individuals with chronic SCI [38]. Other studies have suggested that 25(OH)D status may have an effect on muscle performance and injury prevention, therefore possibly influencing athletic performance [7,39,40]. Foo *et al.* (2009) suggested that poor vitamin D status (<50 nmol/L) was associated with reduced forearm strength (using a handgrip dynamometer) when compared to individuals with vitamin D levels > 50 nmol/L in a group of Chinese adolescent females (*n* = 301) [33]. Although controversial, a recent meta-analysis suggested that vitamin D supplementation had a small, but positive, impact on muscle strength [19]. Researchers have suggested that it may be necessary to increase 25(OH)D levels above 100 nmol/L before a performance benefit can be observed [11,20]. Therefore, further research is warranted to determine the magnitude of effect of vitamin D supplementation on muscle strength and performance in athletes with SCI.

This study is not without limitations, despite the strengths of this well controlled study conducted in elite Paralympic athletes. One limitation of this study is the small sample size when compared to research with an able bodied population. Although our findings may not be generalizable due to the lower power, it should be noted that a sample size of this magnitude in the current study is uncommon for a population of athletes with SCI, but is comparable to the sample sizes used in studies examining able-bodied athletes [9,10]. The ability to detect to detect a small difference in 25(OH)D among indoor and outdoor sports, which is further supported by the low effect sizes, may have been due to the small sample size. As seen in most studies examining athletes with SCI the variation in level of lesion among athletes resulted in differences in physiological capabilities. Furthermore, although there was no correlation observed among geographical location and 25(OH)D, athletes resided in multiple locations throughout the US and Canada. Given there were no differences in 25(OH)D status and geographical location, USA and CAN athletes were analyzed as one group. Although we reported a high proportion of insufficiency and deficiency in our population, since this study did not examine age matched to able-bodied athletes or sedentary subjects with SCI, it is difficult to make comparisons to previously published data in able bodied athletes or sedentary individuals with SCI.

## 5. Conclusions

In conclusion, the findings suggest that a substantial proportion of elite athletes with SCI have low vitamin D status even after the summer months. Contrary to our hypothesis, a seasonal decline in 25(OH)D status was not observed in the current study, regardless of the decrease in sun exposure from autumn to winter. However, it should be noted that a higher proportion of athletes were considered sufficient in autumn compared to winter. In addition, a higher percentage of indoor athletes compared to outdoor athletes were classified as deficient during both the autumn and winter months. Finally, research is warranted to appropriately identify serum 25(OH)D goal levels in athletes with SCI and routine screening and supplementation protocols need to be instituted to prevent vitamin D insufficiencies [7,13].

## Figures and Tables

**Table 1:** Reference 25(OH)D Values.

Category	25(OH) D Level (nmol/L)
Deficient	<50
Insufficient	<80
Sufficient	80–250
Optimal	100–200
High	>250

Reference values from other published studies reporting 25(OH)D concentrations [9,27,29,30,31].

**Table 2:** Descriptive characteristics and seasonal 25(OH)D status organized by indoor and outdoor sport.

	Outdoor		Indoor		
	Athletics	Tennis	Basketball	Rugby	*p*-Value
**Autumn 25(OH)D** **(nmol/L)** (*n* = 39)	76.4 ± 5.2 (*n* = 14)	47.4 (*n* = 1)	70.4 ± 5.7 (*n* = 12)	62.9 ± 5.7 (*n* = 12)	*p* = 0.19
**Winter 25(OH)D** **(nmol/L)** (*n* = 32)	70.4 ± 21.7 (*n* = 13)	49.9 (*n* = 1)	60.2 ± 29.5 (*n* = 10)	74.6 ± 27.5 (*n* = 7)	*p* = 0.75

Data are means ± SD. Note: No differences were found in 25(OH)D between indoor (basketball and rugby) and outdoor sports (athletics and tennis) in the autumn or winter, respectively, using an independent samples *t*-test.

**Table 3:** 25(OH)D status relative to level of Spinal Cord Injury (SCI) lesion.

	C Level	T1–T6	T7–T12	Lumbar	*p*-Value
**Autumn 25(OH)D** **(nmol/L)** (*n* = 39)	58.4 ± 23.7 (*n* = 11)	75.1 ± 18 (*n* = 10)	74.9 ± 16.5 (*n* = 11)	74.4 ± 20.5 (*n* = 5)	*p =* 0.15
**Winter 25(OH)D** **(nmol/L)** (*n* = 32)	73.9 ± 30.2 (*n* = 8)	71.9 ± 28.7 (*n* = 7)	64.9 ± 2.3 (*n* = 11)	62.9 ± 25.5 (*n* = 6)	*p* = 0.59

Data are means ± SD. Note: No differences were found in 25(OH)D between level of SCI lesion in the autumn or winters, respectively, using one-way ANOVA.

**Table 4:** Reported Frequency of Consumption of Dietary Vitamin D by Season.

Food/Vitamin D Content (IU)	Autumn (*n* = 39)	Winter (*n* = 32)
Milk (8 oz), 100 IU		
never, <1/month	9	5
1–2/month	2	5
2–4/week	1	10
5–6/week	6	2
1/day	9	7
2–3/day	4	3
Cereal (6–8 oz), 40 IU		
never, <1/month	11	7
1–3/month	7	12
1/week	1	4
2–4/week	2	6
5–6/week	1	2
1/day	1	1
Fortified Orange Juice (8 oz), 100 IU		
never, <1/month	13	12
1–2/month	7	11
1/week	5	2
2–4/week	7	3
5–6/week	3	3
1/day	2	1
2–3/day	1	0
Egg (1 whole), 18 IU		
never, <1/month	4	5
1–3/month	3	2
1/week	3	3
2–4/week	15	11
5–6/week	6	3
1/day	3	5
2–3/day	3	3
6+/day	1	0
Salmon (3.5 oz), 815 IU		
never, <1/month	10	8
1–3/month	12	13
1/week	5	6
2–4/week	11	5
MVI, 400 IU		
never, <1/month	28	26
1–3/month	1	2
2–4/week	2	0
1/week	0	1
1/day	5	2
2–3/day	1	1

Note: Derived from Halliday *et al.*, 2011 [4].
